# Case Report: Undifferentiated embryonic sarcoma of an adult liver

**DOI:** 10.3389/fonc.2025.1599953

**Published:** 2025-07-25

**Authors:** Shengyong Li, Lexin Bao, Xiujun Li, Liang Bai

**Affiliations:** Department of Hepatobiliary Surgery, Weihai Municipal Hospital, Weihai, China

**Keywords:** undifferentiated embryonal sarcoma, liver, long-term survival, multidisciplinary treatment, adult

## Abstract

Undifferentiated embryonal sarcoma of the liver is a malignant mesenchymal tumor of the liver that predominantly affects children, with exceedingly rare occurrence in adults, and is associated with a poor prognosis. We present a case of undifferentiated embryonal sarcoma of the liver in a previously healthy 45-year-old man admitted to our hospital due to fever. Magnetic resonance imaging revealed a cystic-solid mass in the right lobe of the liver, measuring approximately 15 * 13 * 13 cm in size. Imaging diagnosis raised a suspicion of biliary cystadenocarcinoma with hemorrhage. A hepatic resection was successfully performed, and the histologic diagnosis was undifferentiated embryonal sarcoma of the liver. The patient received a chemotherapy regimen of ifosfamide combined with epirubicin after surgery, and no recurrence was observed after 6 months of follow-up. We herein review the clinical, radiologic, and pathologic features of this rare tumor.

## 1 Introduction

Undifferentiated embryonal sarcoma of the liver (UESL) is a rare and aggressive liver sarcoma originating from the liver’s mesenchymal tissue (1). Most cases occur in children aged 6 to 10 years, and UESL accounts for less than 1% of liver tumors in adults (2, 3). The early symptoms of UESL are similar to those of liver abscess and cystic liver disease, such as abdominal mass, distension, and pain, which are difficult to diagnose clinically (4). Tumor markers such as alpha-fetoprotein (AFP), carbohydrate antigen 19-9 (CA19-9), and carcinoembryonic antigen (CEA) are usually not significantly abnormal (5). The imaging findings of UESL are also non-specific. The right lobe of the liver is a common site of UESL, presenting as a single, large, and well-defined cystic-solid mass (6, 7). Surgical excision and postoperative chemotherapy remain the primary treatments for UESL. However, due to the difficulty of symptom concealment and diagnosis, most patients diagnosed with UESL are in advanced stages of the disease, making them unsuitable for surgical treatment. Therefore, early detection, complete resection, and postoperative adjuvant therapy are the keys to a good prognosis (2, 8).

This case report provides a comprehensive description of the clinical diagnosis and treatment of an adult patient, including imaging, histology, and genetic manifestations, thereby supporting the clinical progress of UESL.

## 2 Case presentation

**Figure 1:**
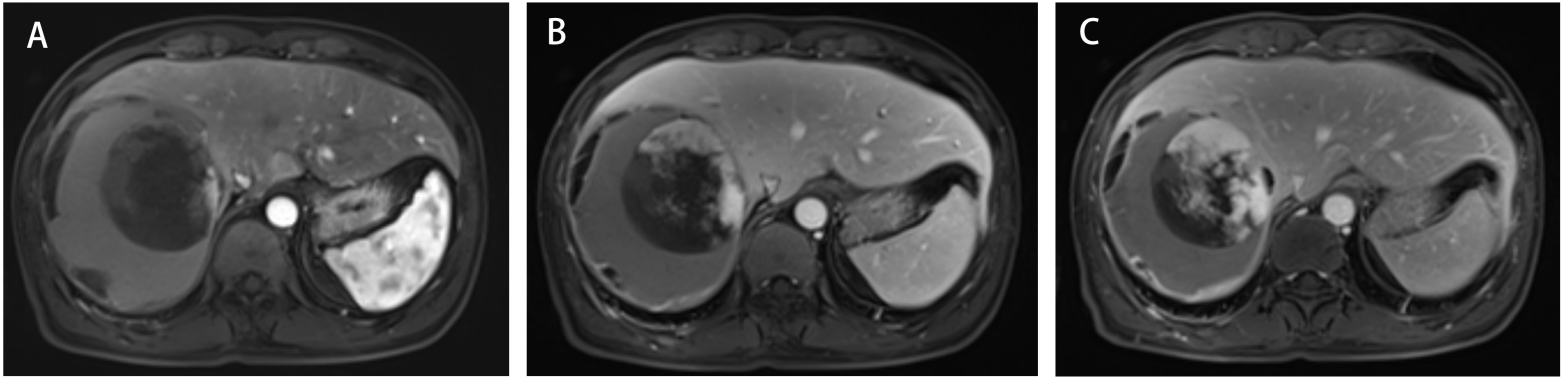
Three-phase enhanced MRI of the abdomen. **(A)** The arterial phase. **(B)** The portal venous phase. **(C)** The delayed phase.

**Figure 2:**
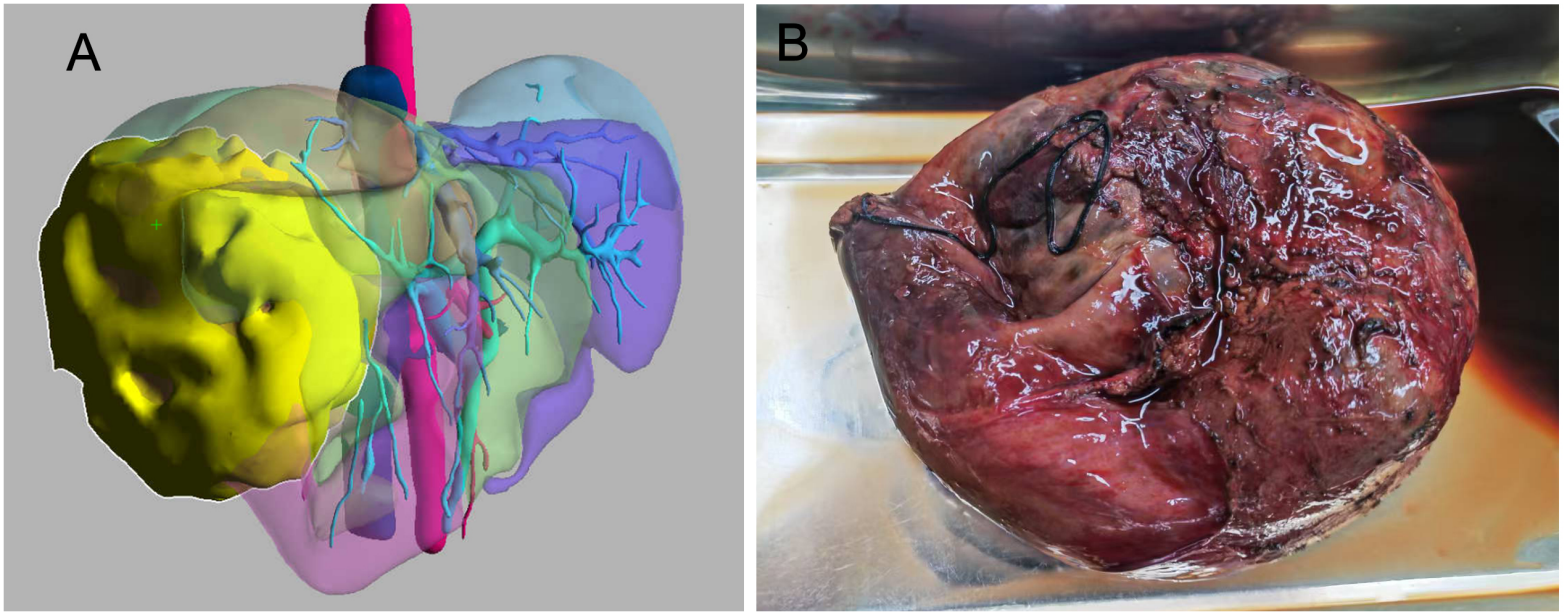
**(A)** Preoperative three-dimensional reconstruction imaging (the lesion is highlighted in yellow). **(B)** Gross photograph of undifferentiated embryonal sarcoma of the liver.

**Figure 3:**
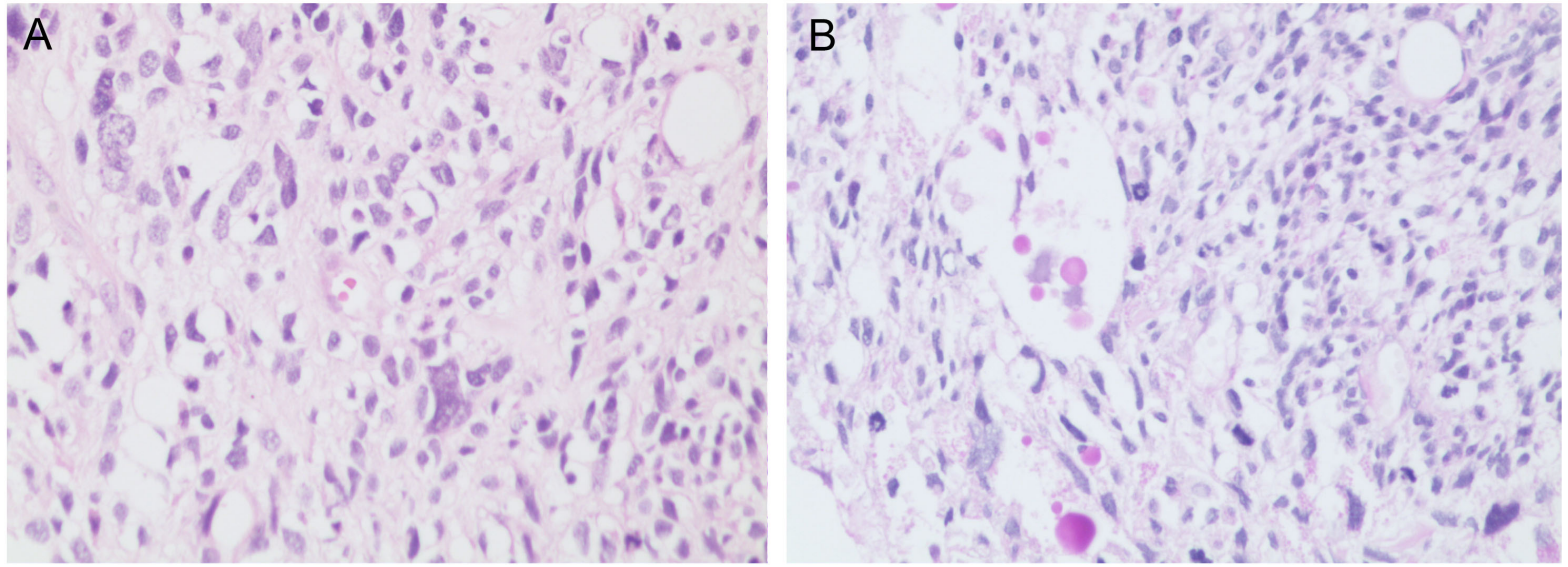
**(A)** The tumor is composed of spindle-shaped cells (×200, H&E stain). **(B)** Some tumor cells contain eosinophilic globules (×200, PAS stain). PAS, Periodic Acid-Schiff.

**Figure 4:**
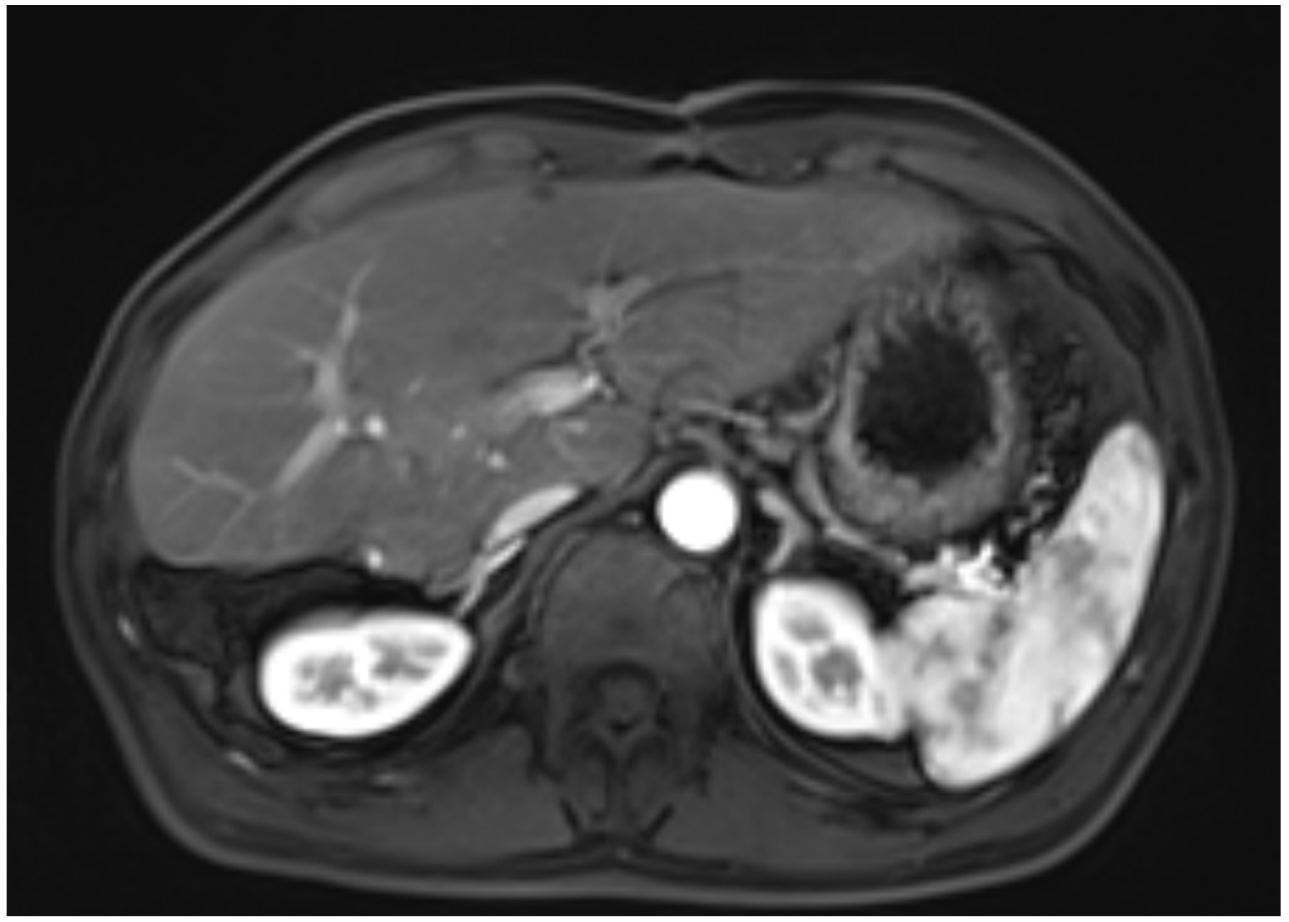
No recurrence was found on MRI 6 months after surgery.

A 45-year-old male patient presented with fever to Weihai Municipal Hospital Affiliated to Shandong University in December 2024. Laboratory examination upon admission showed elevated white blood cell count and hypersensitive C-reactive protein, and no abnormalities were found in the tumor markers (including AFP, CA19-9, and CEA). Physical examination revealed no discernible disparity. The patient had no history of hepatitis B infection, liver cirrhosis, or other malignancies and no family history of disease. Abdominal ultrasound of the patient showed a cystic-solid mass in the right lobe of the liver, measuring approximately 15 * 13 * 13 cm in size, with a thin wall, clear boundary, and internal septa. Color Doppler flow imaging (CDFI) demonstrates sparse peripheral blood flow signals around the nodule. Further enhanced magnetic resonance imaging (MRI) revealed a huge oval lesion in the right lobe of the liver. T1-weighted imaging (T1WI) showed mixed low signal, and T2-weighted imaging (T2WI) showed mixed high signal. The solid part showed diffusion restriction in diffusion-weighted imaging (DWI). After injection of the contrast agent, the solid part of the lesion showed progressive heterogeneous enhancement, especially in the delayed phase (**Figure 1**). Background liver parenchyma was normal without manifestations of fatty liver or cirrhosis. Imaging diagnosis raised a suspicion of biliary cystadenocarcinoma with hemorrhage. Preoperative assessment showed that the patient’s liver function was classified as Child–Pugh A, and the indocyanine green 15-minute retention rate was 3.3%. The standardized future liver remnant (sFLR), calculated from preoperative three-dimensional reconstruction imaging, was 0.88. Therefore, open liver tumor resection was performed under general anesthesia, and a cystic-solid mass originating from the right lobe of the liver was observed, with an intact capsule. The remaining liver tissue appeared reddish and moist, with no cirrhosis or abnormal nodules. The right posterior lobe of the liver along with the mass was completely resected approximately 1 cm from the left margin of the mass (**Figure 2**). The tumor demonstrates a variegated cut surface with gray-red and gray-yellow coloration, showing focal cystic changes filled with clear, straw-colored fluid. Pathological examination revealed a fusiform cell tumor with necrosis, hemorrhage, cystic changes, and mitotic images. Some tumor cells contained eosinophilic bodies, which were positive for Periodic Acid-Schiff (PAS) staining (**Figure 3**). The results of immunohistochemical (IHC) were as follows: Vimentin (+), CK-pan (+), P16 (+), CD34 (+), Desmin (+), H3K27Me3 (+), P53 (missense mutant), Melan A (−), S-100 (−), SMA (−), CD31 (−), MyoD1 (−), Stat6 (−), and Ki-67 labeling index of 65%. Based on the location, morphology, and immunohistochemical results, the diagnosis tended to be UESL of the liver. Postoperatively, the patient’s liver function showed no significant impairment. The patient was discharged on postoperative day 9 following a smooth recovery. Currently, there is no standardized chemotherapy regimen for UESL. Postoperatively, based on multidisciplinary tumor board consensus and published literature (2, 22), the patient received five cycles of combined ifosfamide and epirubicin chemotherapy administered at 21-day intervals (**Table 1**). The chemotherapy regimen was well-tolerated by the patient, who maintained excellent compliance throughout the treatment course. At the 6-month follow-up, hepatic MRI revealed no evidence of tumor recurrence (**Figure 4**).

**Table 1:** Timeline of clinical events.

Date	Clinical event
December 5, 2024	The patient was admitted to the hospital due to febrile condition.
December 7, 2024	Hepatic MRI revealed a huge oval lesion in the right lobe of the liver.
December 12, 2024	The patient underwent partial hepatectomy of the right liver lobe.
December 22, 2024	The patient achieved satisfactory postoperative recovery and was subsequently discharged in stable condition.
January–April 2025	The patient received five cycles of combined ifosfamide and epirubicin chemotherapy.
June 9, 2025	At the 6-month follow-up, hepatic MRI revealed no evidence of tumor recurrence.

## 3 Discussion

UESL is a malignant liver tumor originating from mesenchymal tissue, primarily occurring in children aged 6 to 10 years. It ranks third in incidence among primary malignant liver tumors in children, following hepatoblastoma and hepatocellular carcinoma, and it has also been sporadically reported in adult patients (1, 3). However, this entity is exceptionally rare in adults, constituting less than 1% of primary hepatic neoplasms (9). The non-specific clinical manifestations frequently lead to misdiagnosis and delayed treatment. Adult UESL demonstrates significantly poorer prognosis with a 5-year overall survival (OS) rate of 48.2%, compared to 84.4% in pediatric cases (10, 11).

The clinical symptoms of UESL are insidious, primarily manifesting as abdominal pain or an epigastric mass, with occasional secondary symptoms such as fever, nausea, vomiting, and weight loss. UESL lacks specific serum markers. Although patients with UESL generally do not have underlying liver diseases such as hepatitis or cirrhosis, abnormal liver function may occur due to the compression of surrounding normal liver tissue by the large tumor mass. In cases complicated by intratumoral hemorrhage, necrosis, or infection, elevated leukocytes may be observed (8). Serum tumor markers in UESL patients are typically negative. In this case, laboratory tests, including tumor markers, showed no abnormal findings, which aligns with previous research findings (12, 13). UESL is predominantly located in the right hepatic lobe, and lesions can be detected via ultrasound, computed tomography (CT), or MRI. Typical ultrasound findings reveal a cystic-solid mass with mixed echogenicity, characterized by hyperechoic areas caused by numerous small interfaces within the myxoid matrix (2). In CT images, tumors are displayed as a well-defined unilocular or multilocular hypodense mass. Due to the hydrophilic acidic mucopolysaccharides in the myxoid matrix, which continuously absorb water, CT often indicates fluid density (8, 14). Contrast-enhanced scans show varying degrees of enhancement in the tumor margins and internal solid components. The differences in imaging features between ultrasound and CT can improve preoperative diagnostic accuracy. Notably, the main differential diagnoses for UESL include biliary cystadenocarcinoma, massive hepatocellular carcinoma, mesenchymal hamartoma, and hepatoblastoma. Biliary cystadenocarcinoma predominantly affects middle-aged women, with CT imaging typically revealing multilocular cystic liver lesions characterized by nodular wall projections, irregular wall thickness, and homogeneous enhancement of septations. In contrast, massive hepatocellular carcinoma usually develops in patients with hepatitis-induced cirrhosis, showing significantly elevated AFP levels and CT features of predominantly solid masses containing necrotic areas, demonstrating the classic “wash-in, wash-out” enhancement pattern. Mesenchymal hamartoma occurs primarily in children under 2 years of age, presenting on CT as well-demarcated multilocular cystic lesions with multiple thin septations, smooth walls, and rare soft-tissue components or mural nodules. Hepatoblastoma, mainly affecting children aged 3–5 years without a history of hepatitis or cirrhosis, typically appears on CT as large solid masses frequently exhibiting hemorrhage and necrosis, with amorphous calcifications observed in approximately 50% of cases. While MRI exhibits more distinctive characteristics compared to ultrasound and CT, these imaging modalities are insufficient to differentiate UESL from other space-occupying lesions (7, 13). Preoperative needle biopsy may provide a more definitive diagnosis of UESL, but it carries a risk of tumor spread and should be used cautiously (15, 16). In summary, the preoperative diagnosis of UESL remains challenging, and definitive diagnosis ultimately requires postoperative pathological examination.

The pathological features of UESL include a single, well-demarcated lesion, typically larger than 10 cm, with a fibrous pseudocapsule. The tumor is composed of solid and cystic components. The solid portions exhibit a white or gray-white, fish-flesh-like appearance on cut surfaces, while the cystic areas contain necrotic debris and blood clots (6). Under the microscope, spindle-shaped and stellate tumor cells are diffusely distributed within a myxoid matrix, accompanied by marked cellular atypia and frequent mitotic figures. Additionally, according to the World Health Organization (WHO) classification and diagnostic criteria for tumors of the digestive system, UESL should undergo PAS staining. The presence of PAS-positive eosinophilic bodies within tumor cells and the intercellular stroma serves as a specific pathological indicator of UESL (6). Currently, UESL does not have a specific immunophenotype. Vimentin, CD68, and α1-antitrypsin are typically positive, while the expression of S-100, SMA, Desmin, CD34, Cytokeratin, Myoglobin, and Actin varies (17, 18). These findings suggest that the tumor cells originate from primitive mesenchymal cells. Most immunohistochemical tests are primarily used to exclude other diagnoses. The pathogenesis of UESL is unclear. Given that UESL shares chromosomal abnormalities with mesenchymal hamartoma, some researchers hypothesize that UESL may arise through malignant transformation of mesenchymal hamartoma. Molecular characterization reveals recurrent abnormalities at the 19q13.4 locus, including balanced translocations t(11;19)(q13;q13.4) and t(15;19)(q15;q13.4). Tumor protein 53 (TP53) mutations have also been identified in a subset of cases. TP53 is a tumor suppressor gene whose mutations lead to loss of apoptotic control and impaired DNA repair, thereby increasing the risk of tumorigenesis and progression. In this case, the tumor cells demonstrated diffusely strong positive expression of TP53. However, definitive genetic testing could not be performed due to the patient’s financial constraints (20, 21).

UESL is a highly malignant tumor characterized by rapid progression and aggressive invasiveness. It is often diagnosed at an advanced stage with a large tumor volume. Historically, treatment with surgical resection alone yielded poor outcomes, with high rates of postoperative recurrence and metastasis, resulting in a 5-year survival rate below 37.5% (8, 19). In recent years, advancements in comprehensive cancer therapy have significantly improved the prognosis of UESL patients through a combination of surgical resection and postoperative adjuvant chemotherapy. Although no standardized chemotherapy regimen exists, multiple studies have demonstrated the efficacy of ifosfamide combined with epirubicin in treating UESL, markedly enhancing overall survival rates (22). The patient in this case was treated with this chemotherapy regimen and showed no signs of tumor recurrence or metastasis during a 6-month follow-up. However, the 6-month follow-up period is insufficient to draw meaningful conclusions regarding long-term prognosis. We will continue to monitor this patient’s clinical course to generate more robust survival data for UESL treatment evaluation.

## 4 Conclusion

Given the insidious clinical presentation of UESL, the patient already had a large tumor volume at initial diagnosis. However, through aggressive surgical intervention followed by adjuvant chemotherapy, favorable short-term outcomes were achieved. The preoperative diagnosis of UESL remains challenging, with high misdiagnosis rates. For suspected cases, multidisciplinary collaboration is strongly recommended. Complete tumor resection combined with systemic therapy appears crucial for long-term survival, although the optimal treatment standards and efficacy require further investigation through multicenter studies.

## Data Availability

The raw data supporting the conclusions of this article will be made available by the authors, without undue reservation.

## References

[1] Shi Y Rojas Y Zhang W Beierle EA Doski JJ Goldfarb M. Characteristics and outcomes in children with undifferentiated embryonal sarcoma of the liver: A report from the National Cancer Database. Pediatr Blood Cancer. (2017) 64:e26272. doi: 10.1002/pbc.26272, PMID: 27781381 PMC5333454

[2] Huang Y He X Shangguan L Zhou Y Zhang L Wu Y. Undifferentiated embryonal sarcoma of the liver masquerading as a cystadenoma in a young adult: a case report. J Med Case Rep. (2024) 18:517. doi: 10.1186/s13256-024-04867-8, PMID: 39487550 PMC11531131

[3] Weinberg AG Finegold MJ. Primary hepatic tumors of childhood. Hum Pathol. (1983) 14:512–37. doi: 10.1016/s0046-8177(83)80005-7, PMID: 6303939

[4] Shu B Gong L Zhuang X Cao L Yan Z Yang S. Undifferentiated embryonal sarcoma of the liver in adults: Retrospective analysis of a case series and systematic review. Oncol Lett. (2020) 20:102. doi: 10.3892/ol.2020.11963, PMID: 32831921 PMC7439129

[5] Lin W-Y Wu K-H Chen C-Y Guo B-C Chang Y-J Lin M-J. Treatment of undifferentiated embryonal sarcoma of the liver in children. Cancers. (2024) 16:897. doi: 10.3390/cancers16050897, PMID: 38473259 PMC10931367

[6] Putra J Ornvold K. Undifferentiated embryonal sarcoma of the liver: A concise review. Arch Pathol Lab Med. (2015) 139:269–73. doi: 10.5858/arpa.2013-0463-RS, PMID: 25611111

[7] Jiang P Jiao Y Niu CY Liu YH. Undifferentiated embryonal sarcoma of the liver with epithelioid features in an adult patient. Medicine. (2021) 100:e28265. doi: 10.1097/MD.0000000000028265, PMID: 34918699 PMC10545351

[8] Zhang C Jia CJ Xu C Sheng QJ Dou XG Ding Y. Undifferentiated embryonal sarcoma of the liver: Clinical characteristics and outcomes. World J Clin cases. (2020) 8:4763–72. doi: 10.12998/wjcc.v8.i20.4763, PMID: 33195644 PMC7642548

[9] Noguchi K Yokoo H Nakanishi K Kakisaka T Tsuruga Y Kamachi H. A long-term survival case of adult undifferentiated embryonal sarcoma of liver. World J Surg Oncol. (2012) 10:65. doi: 10.1186/1477-7819-10-65, PMID: 22540346 PMC3407002

[10] Ziogas IA Zamora IJ Lovvorn Iii HN Bailey CE Alexopoulos SP. Undifferentiated embryonal sarcoma of the liver in children versus adults: A national cancer database analysis. Cancers. (2021) 13:2918. doi: 10.3390/cancers13122918, PMID: 34208030 PMC8230649

[11] Endo YK Fujio A Murakami K Sasaki K Miyazawa K Kashiwadate T. Long-term survival of an adult patient with undifferentiated embryonal sarcoma of the liver with multidisciplinary treatment: a case report and literature review. Surg Case Rep. (2022) 8:85. doi: 10.1186/s40792-022-01436-3, PMID: 35508823 PMC9068849

[12] Chen JH Lee CH Wei CK Chang SM Yin WY. Undifferentiated embryonal sarcoma of the liver with focal osteoid picture—A case report. Asian J Surg. (2013) 36:174–8. doi: 10.1016/j.asjsur.2012.06.012, PMID: 24054758

[13] Li X-W. Undifferentiated liver embryonal sarcoma in adults: A report of four cases and literature review. World J Gastroenterol. (2010) 16:4725–32. doi: 10.3748/wjg.v16.i37.4725, PMID: 20872975 PMC2951525

[14] Moon WK Kim WS Kim IO Yeon KM Yu IK Choi BI. Undifferentiated embryonal sarcoma of the liver: US and CT findings. Pediatr Radiol. (1994) 24:500–3. doi: 10.1007/BF02015012, PMID: 7885785

[15] Naveen K Sameer V Palash JD Kochhar R Srinivasan R Khandelwal N. Undifferentiated embryonal sarcoma of liver in an adult masquerading as complicated hydatid cyst. Ann Hepatol. (2011) 10:81–3. doi: 10.1016/S1665-2681(19)31592-3, PMID: 21301015

[16] Geel JA Loveland JA Pitcher GJ Beale P Kotzen J Poole JE. Management of undifferentiated embryonal sarcoma of the liver in children: A case series and management review. South Afr Med J. (2013) 103:728–31. doi: 10.7196/samj.6058, PMID: 24079623

[17] Kiani B Ferrell LD Qualman S Frankel WL. Immunohistochemical analysis of embryonal sarcoma of the liver. Appl Immunohistochem Mol Morphol. (2006) 14:193–7. doi: 10.1097/01.pai.0000173052.37673.95, PMID: 16785789

[18] Zheng JM Tao X Xu AM Chen XF Wu MC Zhang SH. Primary and recurrent embryonal sarcoma of the liver: clinicopathological and immunohistochemical analysis. Histopathology. (2007) 51:195–203. doi: 10.1111/j.1365-2559.2007.02746.x, PMID: 17573940

[19] Cao Q Ye Z Chen S Liu N Li S Liu F. Undifferentiated embryonal sarcoma of liver: a multi-institutional experience with 9 cases. Int J Clin Exp Pathol. (2014) 7:8647–56., PMID: 25674229 PMC4313983

[20] Mathews J Duncavage EJ Pfeifer JD. Characterization of translocations in mesenchymal hamartoma and undifferentiated embryonal sarcoma of the liver. Exp Mol Pathol. (2013) 95:319–24. doi: 10.1016/j.yexmp.2013.09.006, PMID: 24120702

[21] Setty BA Jinesh GG Arnold M Pettersson F Cheng CH Cen L. The genomic landscape of undifferentiated embryonal sarcoma of the liver is typified by C19MC structural rearrangement and overexpression combined with TP53 mutation or loss. PLoS Genet. (2020) 16:e1008642. doi: 10.1371/journal.pgen.1008642, PMID: 32310940 PMC7192511

[22] Techavichit P Masand PM Himes RW Abbas R Goss JA Vasudevan SA. Undifferentiated embryonal sarcoma of the liver (UESL): A single-center experience and review of the literature. J Pediatr Hematol Oncol. (2016) 38:261–8. doi: 10.1097/MPH.0000000000000529, PMID: 26925712

